# Insights into the use of two novel supramolecular compounds as corrosion inhibitors for stainless steel in a chloride environment: experimental as well as theoretical investigation

**DOI:** 10.1039/d3ra07397a

**Published:** 2023-12-04

**Authors:** A. S. Fouda, S. E. H. Etaiw, A. M. Ibrahim, A. A. El-Hossiany

**Affiliations:** a Department of Chemistry, Faculty of Science, Mansoura University Mansoura 35516 Egypt asfouda@hotmail.com +2 050 2202264 +2 050 2365730; b Department of Chemistry, Faculty of Science, Tanta University Tanta Egypt; c Delta for Fertilizers and Chemical Industries Talkha Egypt

## Abstract

Novel supramolecular (SCPs) compounds such as: {[Ni (EIN)_4_(NCS)_2_]}, SCP1 and {[Co (EIN)_4_ (NCS)_2_]}, SCP2 have been studied using weight loss (WL) and electrochemical tests on the corrosion performance of stainless steel 304 (SS304) in 1.0 M hydrochloric acid (HCl) solution. The experimental results revealed that inhibition efficacy (*η*%) rises with increasing concentrations of SCPs and reached 92.3% and 89.6% at 16 × 10^−6^ M, 25 °C, from the WL method for SCP1 and SCP2, respectively. However, by raising the temperature, *η*% was reduced. Polarization measurements (PDP) showed that the SCPs molecules represent a mixed-type. The SCPs were adsorbed on a SS304 surface physically, and the Langmuir adsorption isotherm was found to govern the adsorption process. The determination of thermodynamic parameters was carried out at various temperatures. Quantum chemical calculations were calculated to prove the adsorption process of SCP components, using the molecular dynamics (MD) simulations and electron density map. The inhibition performance of SCPs for SS304 dissolution in an acidic medium was proved to be excellent through FT-IR and AFM analysis. The results obtained from all measurements exhibit a high level of agreement with each other.

## Abstract 2

Novel supramolecular (SCPs) compounds such as SCP1 and SCP2 have been studied using WL, PDP and EIS tests on the corrosion performance of SS304 in 1.0 M HCl solution.

## Graphical abstract

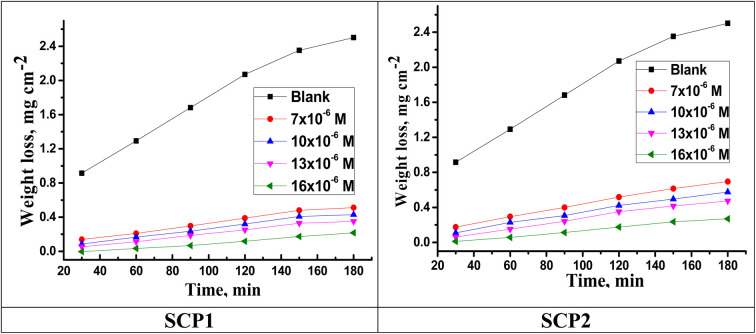

## 1. Introduction

The pickling process is an important step in the metal finishing industry. Hydrochloric acid is usually used as a method for achieving this goal.^1,2^ There are a number of financial and environmental risks associated with corrosion of metallic equipment in pickling.^3^ The high corrosion resistance of stainless steel makes it ideal for a variety of industrial applications. There is a strong passive film, which is formed by the mixture of iron oxide and chromium oxyhydroxide on the outer surface.^4^ Stainless steel surfaces can be reduced in corrosion resistance by chlorine-ion-containing solutions.^5^ It is true that part of the passive film is attacked by attacking ions, and other parts remain unharmed, so a galvanic couple forms on the steel surface, increasing corrosion. This is called pitting corrosion.^6^ The study of inhibitors to prevent corrosion of metals, especially stainless steel, has been conducted in the past few decades.^7–9^ Corrosion inhibitors are commonly used to defend these alloys from corrosion when operating or cleaning them.^10^ In the literature, there are numerous corrosion inhibitors for protecting metal specimens from acidic, salty, and similar harsh environments.^11^ The coordination complexes are excellent corrosion inhibitors because of their multifunctional structure, heteroaromatic chemistry, and richness in π-systems.^12^ Because organic linkers are a common feature of these nanosized composites, researchers have been studying them for the past three decades owing to their ability to create a variety of structures.^13,14^ In various aqueous solutions, supramolecular complex composites have been demonstrated to inhibit corrosion of several metals.^15^ Aluminium terephthalates and their nanocomposite have been used as corrosion inhibitors against aluminium alloy corrosion in ethylene glycol solutions containing chloride ions, for instance.^16^ Nanoparticles improved inhibition efficiency from 86.52% to 90.8%, according to the results obtained. There have been reports of anticorrosion evidence for a variety of supramolecular chemical types. Based on the findings, this study suggests that corrosion inhibitors might be improved with a variety of benefits, including uniqueness and effectiveness. In this study, supramolecular nanosized complexes were synthesized by incorporating nickel and cobalt nanoparticles. Tests were conducted using 1.0 M HCl solution to assess the anticorrosion activity of the supramolecular nanoscale composite (SCP nanoscale).^17^ The porous and crystalline surfaces of these materials make them easy to adsorb.^18^ SCPs have been discovered to be efficient corrosion inhibitors due to their heteroaromatic ligands.^19^ This study examined synthesized and characterized SCP1 and SCP2 by single crystal structure and thermal analysis with a variety of methods including WL procedures, PDP and EIS tests. The inhibitors' effects on the SS304 surface were also determined using AFM analysis in HCl. Additionally, electron densities were calculated theoretically.

## 2. Experiments

### 2.1 Synthesis of inhibitors

#### 2.1.1 Synthesis of {[Ni (EIN)_4_(NCS)_2_]}, SCP1

The SCP1 results by mixing amount of molar ratio 1 : 4 : 2 of Ni (NO_3_)_2_·6H_2_O in 10 mL H_2_O, EIN in 10 mL CH_3_CN and KSCN in 5 mL water at ambient temperature. The resultant mixture was stirred gently for 20 min at room temperature. After about 3 days, green prismatic crystals of SCP1 were obtained. After filtration, washing with small quantities of cold H_2_O and CH_3_CN and overnight drying, (81%) of SCP1 had been obtained.^20^ Elemental analysis yielded the following results for SCP1 (C_34_H_36_NiN_6_O_8_S_2_); C, 52.39; H, 4.65; N, 10.78%. Found: C, 52.33; H, 4.68; N, 10.81%.

#### 2.1.2 Synthesis of {[Co(EIN)_4_ (NCS)_2_]}, SCP2

At ambient temperature, a solution containing 1 mmol of cobalt nitrate hydrate (Co(ii), 0.291 g) dissolved in 20 mL of deionized water was slowly added to a stirred solution of 2 mmol (0.302 g) of EIN. The mixture obtained was stirred for a short duration, after which 5 mL of a water solution containing 2 mmol (0.194 g) of potassium thiocyanate was added drop by drop to the mixture, followed by additional stirring for a few minutes. Afterward, the solution was allowed to undergo gradual evaporation over a period of one month, resulting in the formation of pink crystals for SCP2 (436 mg, 63% yield).^21^ Elemental analysis yielded the following results for SCP2 (C_34_H_36_CoN_6_O_8_S_2_): calculated: C, 52.37; H, 4.65; N, 10.78%. Experimental: C, 52.41; H, 4.68; N, 10.82% as shown in Table 1. All chemicals used are of BDH grade and used as received without further purification.

**Table 1:** The molecular structure, weight, and formula of SCPs

SCPs	SCP1	SCP2
Mol. structure	Ni(EIN)_4_(NCS)_2_	Co(EIN)_4_(NCS)_2_
Mol. formula	C_34_H_36_N_6_NiO_8_S_2_	C_34_H_36_N_6_O_8_S_2_Co
Mol. weight	779.53	779.76
Structure	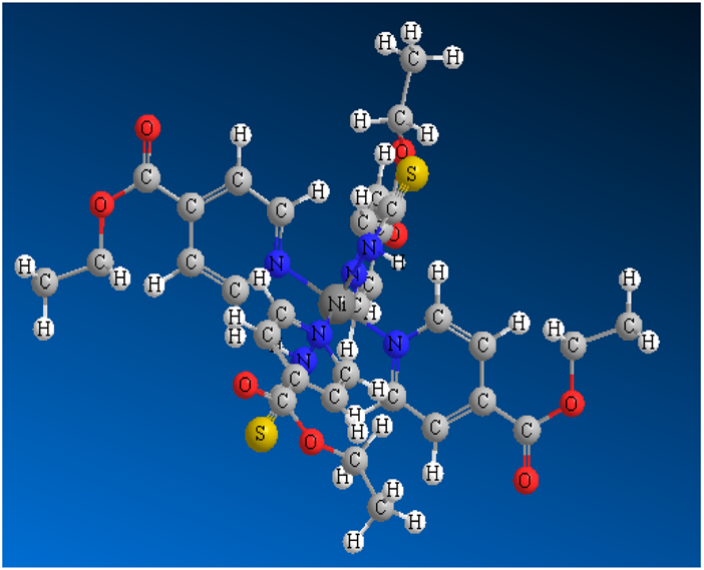	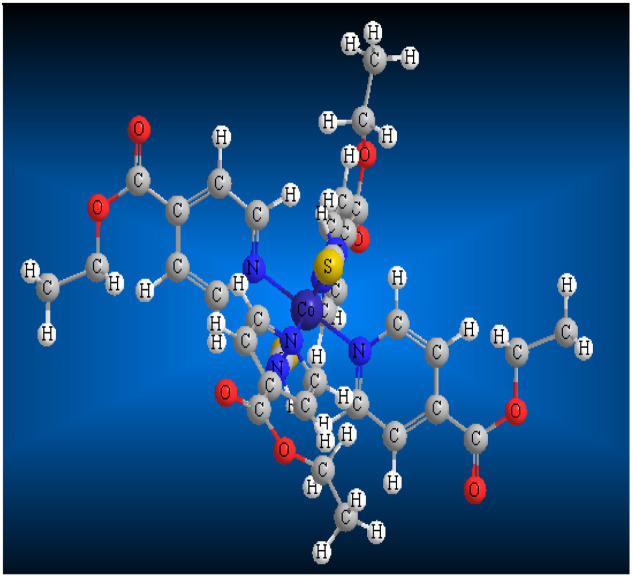

### 2.2 Specimens and test solutions

In this study, the sample of SS304 which used for WL and electrochemical measurements contains C 0.03%, Mn 2%, P 0.045%, N 0.01%, Si 75%, Cr 18–20%, Ni 8–12%, and Fe rest of the sample. Each test was repeated three times to ensure reliable and reproducible results. The corrosion tests in this study were conducted with a solution of hydrochloric acid (1.0 M HCl) obtained by diluting 37% HCl provided by Merck in bidistilled water. As an inhibitory solution, was diluted with 1.0 M HCl solution. A very small portion of dimethyl sulfooxide (DMSO) (5 mL) was added to the blank solution, and the compounds had excellent solubility in HCl solution. The various concentrations of SCPs were in the range of 7 × 10^−6^ M to 16 × 10^−6^ M and prepared from the stock solution (1 × 10^−3^ M) by dilution with 1 M HCl solution.

### 2.3 WL method

Based on the following procedures, the WL of SS304 samples was calculated: (1) weighing polished samples before dipping in the corrosive solution, (2) immersing them in corrosion inhibitors and without corrosion inhibitors, (3) removing, cleaning and drying them with cool air, (4) reweighing the required samples. Using WL, corrosion rate and inhibition efficiency (%*η*_W_) could be calculated as follows from eqn (1) and (2):^22^1
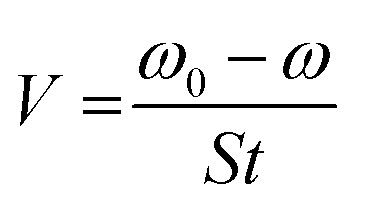
2
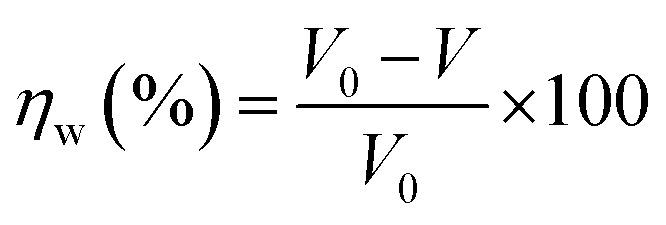


The WL of SS304 without and with supramolecular (SCPs) inhibitors are represented by *ω*_0_ and *ω*, the corrosion rate is denoted by *V*_0_ while the exposed electrode surface area is denoted by *S* and *t* is the time of dipping (min). Thus, *V*_0_ and *V* correspond to the average corrosion rates of SS304 without and with SCPs inhibitors. Under every condition, seven parallel samples were used for the WL experiments.

### 2.4 Electrochemical techniques

An electrochemical reaction was studied with a Potentiostat/Galvanostat/ZRA and Gamry Instrument Series G 750™ analysis software. Three electrodes were used in all electrochemical testing. A reference electrode is made up of Ag/AgCl. Working electrode was SS304 specimen, with exposed area of 1 cm^2^, and counter electrode was platinum electrode.^23^ A 30 minute stabilization step was performed on the electrode immersed in the test solution before each electrochemical test. Tests performed on polarization were done at a scan speed of 0.2 mV s^−1^ at ±250 mV *vs.* open potential circuit (OCP). AC signals of amplitude 10 mV at previous OCP potential were used for electrochemical impedance spectroscopy (EIS) in the frequency range 100 kHz to 10 mHz. As a mean of evaluating corrosion performance, Nyquist and Bode diagrams were used.

### 2.5 Theoretical studies

#### 2.5.1 Quantum chemical calculations

The DMol3 module created in Materials Studio version 7.0 and Gaussian 0.9 software were used in theoretical simulations to evaluate the relationship between the molecular structure and the reactivity of SCPs compounds.^24^

#### 2.5.2 Monte-Carlo simulations (MC)

Using MC, the optimal positioning of SCPs inhibitors on the apparent of SS (111) was evaluated. It is believed that the SS (111) crystal surface used in this simulation is to its most stable according to the literature.^25,26^ The estimation module was initially used to carry out the geometrical optimization of water and the inhibitor molecule. Compass stimulation along with force field were implemented to SCP on SS (111) optimized surface. The substrate–adsorbate system configuration space was searched using the Monte-Carlo approach to identify low-energy adsorption sites where the temperature gradually decreases.^27^

### 2.6 Surface morphology

Scanning probe microscopy (SPM) is used to study the SS304 surface using the Atomic force microscopy (AFM) apparatus (Model nanosurf c3000), with greater concentrations of the SCPs and without SCPs for 24 h, dipping period with confirmed resolution in the region of fractions of a manometer.^28^ The AFM parameters are: mode: BudgestSensors Tap190AL-G Sillicon, AFM probe with long Cantilever, resonant freq.: 190 kHz, force constant: 20 nN, coating: reflective Al, AFM tip shape: rotated, scan rate 1 Hz.

### 2.7 Fourier transfer infrared spectroscopy (FTIR) analysis

The two synthesised SCPs compounds showed distinctive bands in the FTIR spectrum. “FTIR was carried out using a Thermo Fisher Nicolet IS10, USA spectrophotometer in the presence of (KBr)”, which was used to verify and confirm the chemical structure for the substances.^29^

## 3. Results and discussion

### 3.1 WL analysis

**Fig. 1:**
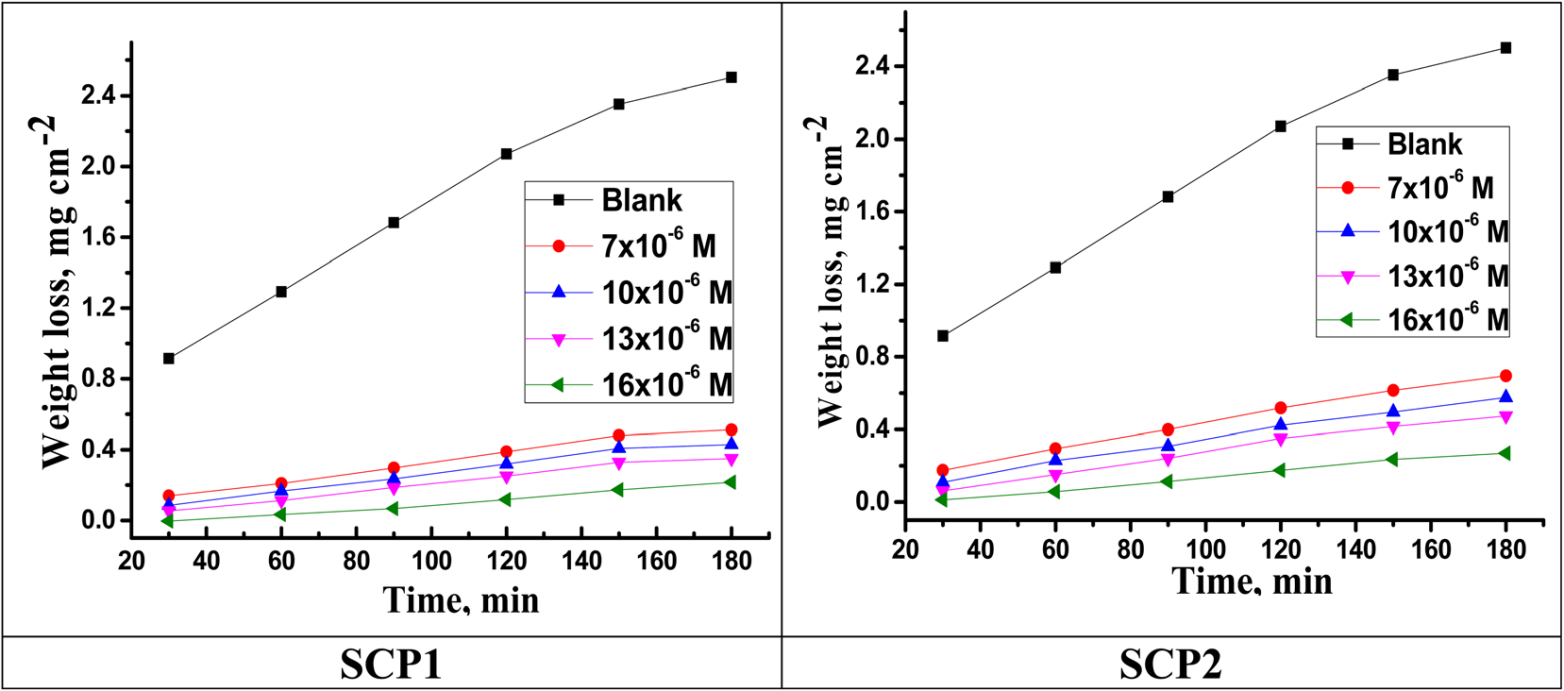
Time-WL bends of SS304 in HCl with and without various concentrations of SCPs inhibitors at 25 °C.

Fig. 1 shows the WL time curves for SS304 corrosion with and without different concentrations of SCPs inhibitors. As shown in the figure, the curves in the presence of different concentrations of SCPs are below the curves in their absence, indicating the adsorption of inhibitory molecules on the surface of SS304 and therefore (*η*%) rises. Corrosion rate (*k*_corr_) and inhibitor efficacy (*η*%) of SCPs inhibitors were calculated for SS304 according to the eqn (1) and (2) under different temperatures, and are reported in Table 2. At high temperature, corrosion of SS304 increases under all conditions. According to this theory, the *k*_corr_ constant rises at a relatively higher temperature as shown in Table 3, which accelerates the electrochemical reactions.^30^ The desorption of corrosion inhibitor molecules may occur at high temperatures, accelerating further corrosion.^31^

**Table 2:** *η*% at various concentrations of SCPs and altered temperatures for SS304 corrosion at 120 min in 1.0 M HCl solution

Comp.	Conc., ×10^6^ M	*η* (%)				
		25 °C	30 °C	35 °C	40 °C	45 °C
SCP1	7	85.0 ± 0.1202	82.8 ± 0.0667	81.0 ± 0.133333	78.9 ± 0.1732	78.4 ± 0.1202
	10	87.2 ± 0.0882	85.9 ± 0.1732	84.3 ± 0.145297	82.3 ± 0.1155	81.6 ± 0.1155
	13	89.5 ± 0.2309	88.3 ± 0.1764	86.6 ± 0.088192	85.0 ± 0.1000	85.3 ± 0.0882
	16	92.5 ± 0.1453	90.8 ± 0.0882	88.9 ± 0.066667	87.8 ± 0.1453	87.0 ± 0.1764
SCP2	7	74.7 ± 0.1155	73.9 ± 0.1202	72.5 ± 0.233333	68.9 ± 0.3283	68.2 ± 0.1856
	10	80.1 ± 0.0577	79.3 ± 0.1155	76.9 ± 0.11547	73.6 ± 0.0882	72.6 ± 0.2028
	13	83.7 ± 0.1453	82.8 ± 0.1202	81.0 ± 0.24037	78.7 ± 0.1155	78.1 ± 0.1202
	16	86.4 ± 0.1202	85.8 ± 0.0882	84.1 ± 0.176383	81.8 ± 0.1528	80.3 ± 0.1528

**Table 3:** Corrosion rate (*k*) at various concentrations of SCPs and altered temperatures for SS304 corrosion at 120 min in 1.0 M HCl solution

Comp.	Conc., ×10^6^ M	*k*				
		25 °C	30 °C	35 °C	40 °C	45 °C
Blank		0.133	0.163	0.216	0.441	0.564
SCP1	7	0.021 ± 0.0013	0.028 ± 0.0012	0.041 ± 0.0017	0.093 ± 0.0002	0.122 ± 0.0012
	10	0.017 ± 0.0015	0.023 ± 0.0014	0.034 ± 0.0013	0.078 ± 0.0011	0.104 ± 0.0017
	13	0.014 ± 0.0017	0.019 ± 0.0017	0.029 ± 0.0015	0.066 ± 0.0017	0.083 ± 0.0011
	16	0.010 ± 0.0014	0.015 ± 0.0018	0.024 ± 0.0014	0.054 ± 0.0015	0.073 ± 0.0012
SCP2	7	0.033 ± 0.0015	0.042 ± 0.0016	0.059 ± 0.0017	0.137 ± 0.0014	0.179 ± 0.0013
	10	0.026 ± 0.0013	0.033 ± 0.0013	0.049 ± 0.0016	0.116 ± 0.0013	0.154 ± 0.0017
	13	0.021 ± 0.0012	0.028 ± 0.0011	0.041 ± 0.0014	0.093 ± 0.0011	0.123 ± 0.0016
	16	0.018 ± 0.0010	0.023 ± 0.0013	0.034 ± 0.0011	0.080 ± 0.0012	0.111 ± 0.0012

### 3.2 Adsorption isotherm behaviour

**Fig. 2:**
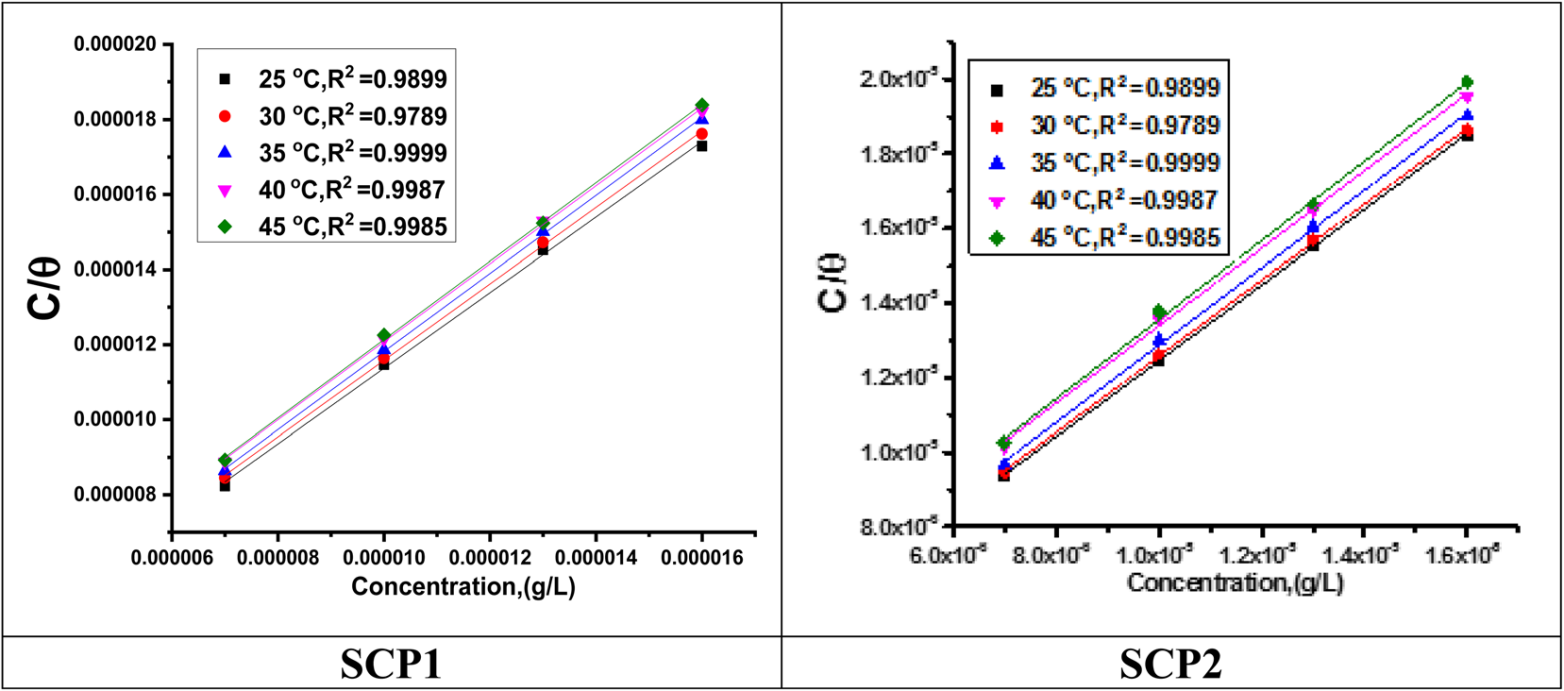
Langmuir diagrams of inhibitor SCP1 and SCP2 on SS304 surface in 1.0 M HCl.

Observation and analysis of the better adsorption isotherms of synthesized SCPs inhibitors using Langmuir, Flory-Huggins, Temkin, Frumkin, Freundlich type, and Kinetic model showed that the inhibition of adsorption behaviour on SS304 surfaces followed the Langmuir isotherm (Fig. 2)^32^ due to: (1) the straight lines with slopes close to unity on the plot of *C*/*vs. C* (concentration) at different temperatures; and (2) the good correlation (*R*^2^ > 0.99), which was utilized to choose the isotherm that best suited the data. According to experimental data, SCPs adhered to this isotherm when adhering to the surface of SS304. According to this isotherm, there are no interactions between the adsorbed species and they each occupy a single location.^33^Eqn (3) provides the isotherm:^34^3
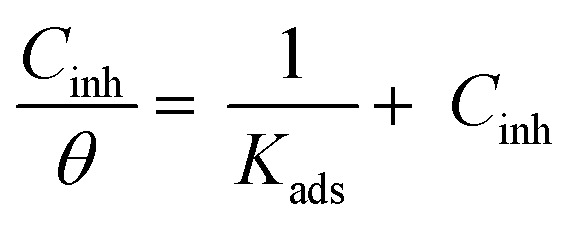


*K*_ads_ represents the equilibrium constant of SS304 substrate adsorption, and *C*_inh_ represents the molar weight of the synthesized SCPs inhibitors. The standard free energy of adsorption 
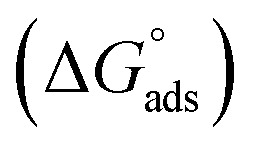
 calculated from (*K*_ads_) according eqn (4):^35^4
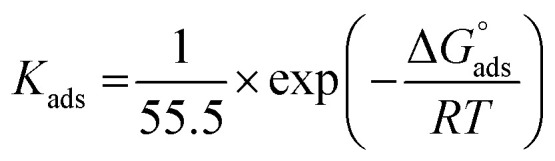


The water concentration at the interface among the SS304 and the solution is 55.5 (mol L^−1^). Table 4 lists the 
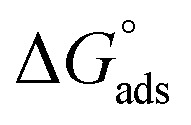
 values. If 
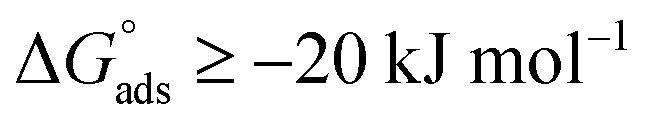
 and 
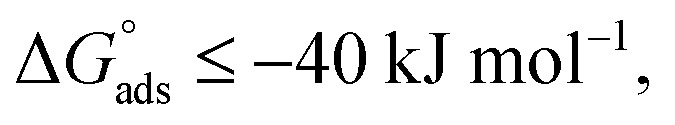
 then according to literature it is physisorption or chemisorption.^36^ The calculated 
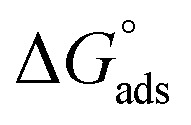
 values are between −23.1 and −21.5 kJ mol^−1^. We can therefore conclude that the adsorption is mixed (physical or chemical) based on the obtained values of the 
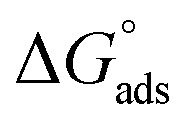
 (between 20 and 40 kJ mol^−1^),^37^ but mainly physical based on the values of 
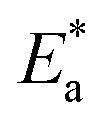
 and *η*%. By using the Vant's Hoff equation, the enthalpy of adsorption 
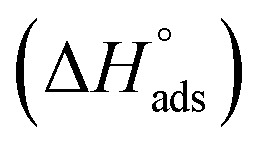
 was determined as follows from eqn (5):^38^5
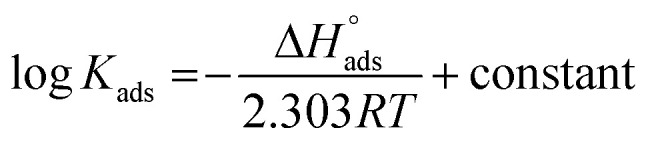


**Table 4:** Thermodynamic parameters obtained from Langmuir adsorption isotherm

Inh.	Temp., °C	*R* ^2^	*K*_ads_, M^−1^	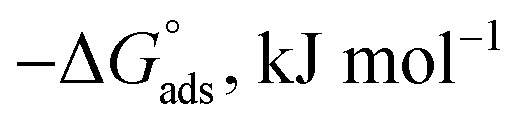	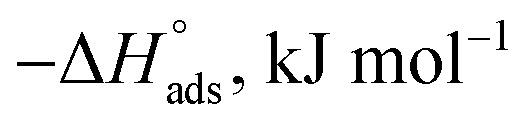	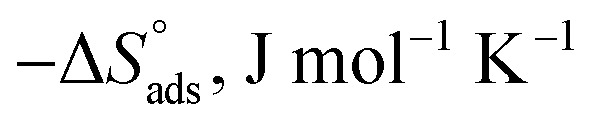
SCP1	25	0.998	198	23.1	33.1	77.3
	30	0.997	166	22.9		75.7
	35	0.996	135	22.8		74.1
	40	0.999	105	22.6		71.9
	45	0.995	87	22.4		70.4
SCP2	25	0.998	143	22.2	31.8	74.5
	30	0.999	110	21.9		72.3
	35	0.998	88	21.7		70.5
	40	0.997	73	21.6		68.9
	45	0.999	62	21.5		67.6

**Fig. 3:**
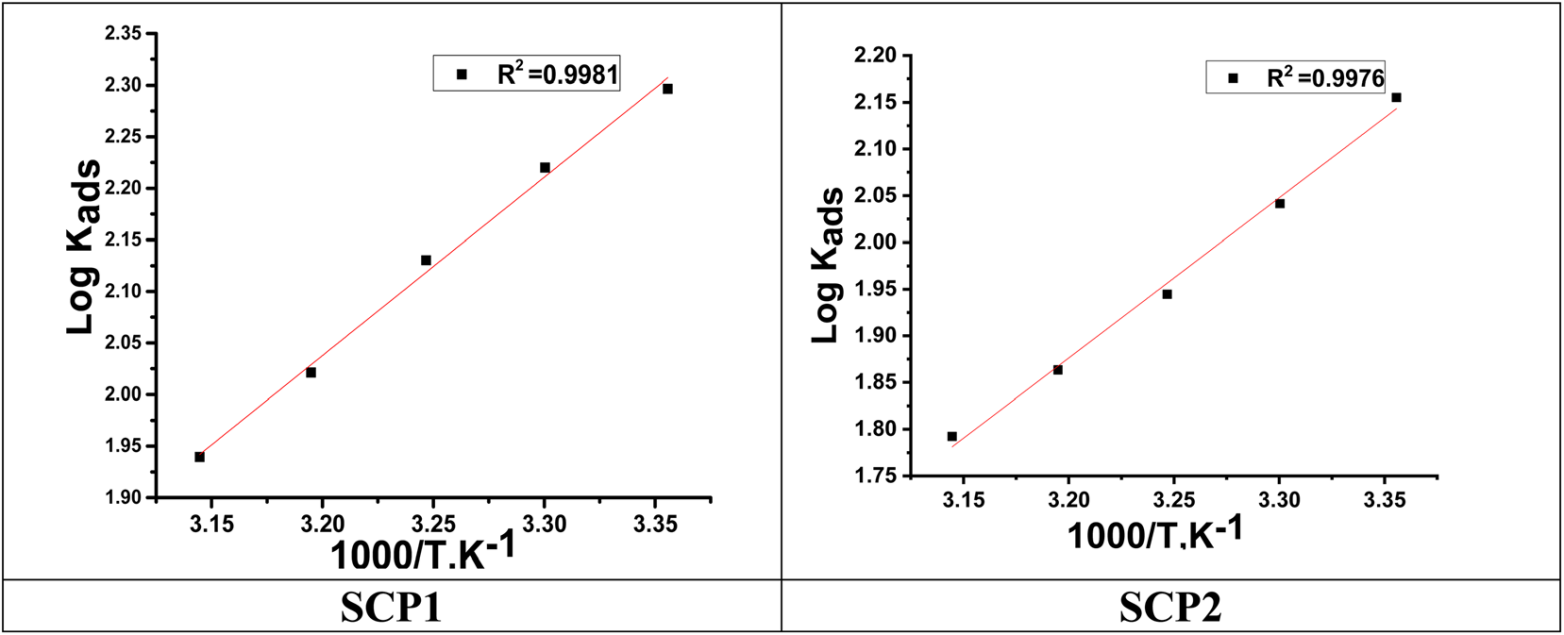
The relation between log *K*_ads_ and 1000/*T* curve for the dissolution of SS304 in 1.0 M HCl in the presence of SCPs compounds.

Fig. 3 indicates plots of log *K*_ads_ and 1/*T*. 
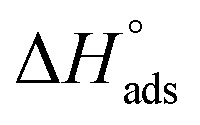
 can be determined from the line slope. The entropy 
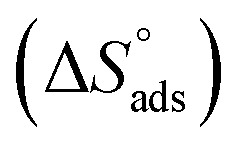
 of adsorption can be acquired by employing the following eqn (6):6
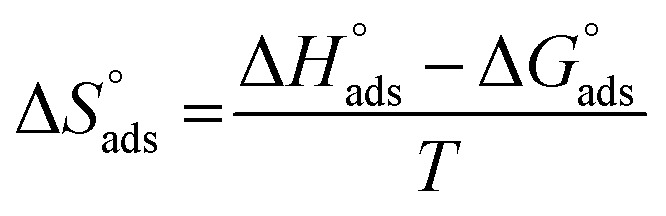


A negative value of 
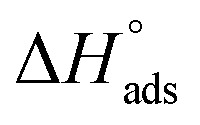
 indicates an exothermic adsorption process. The 
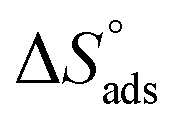
 have negative sign, indicating that there is an increase in ordering during the process of adsorption as shown in Table 4.^39^

### 3.3 Kinetic parameters

**Fig. 4:**
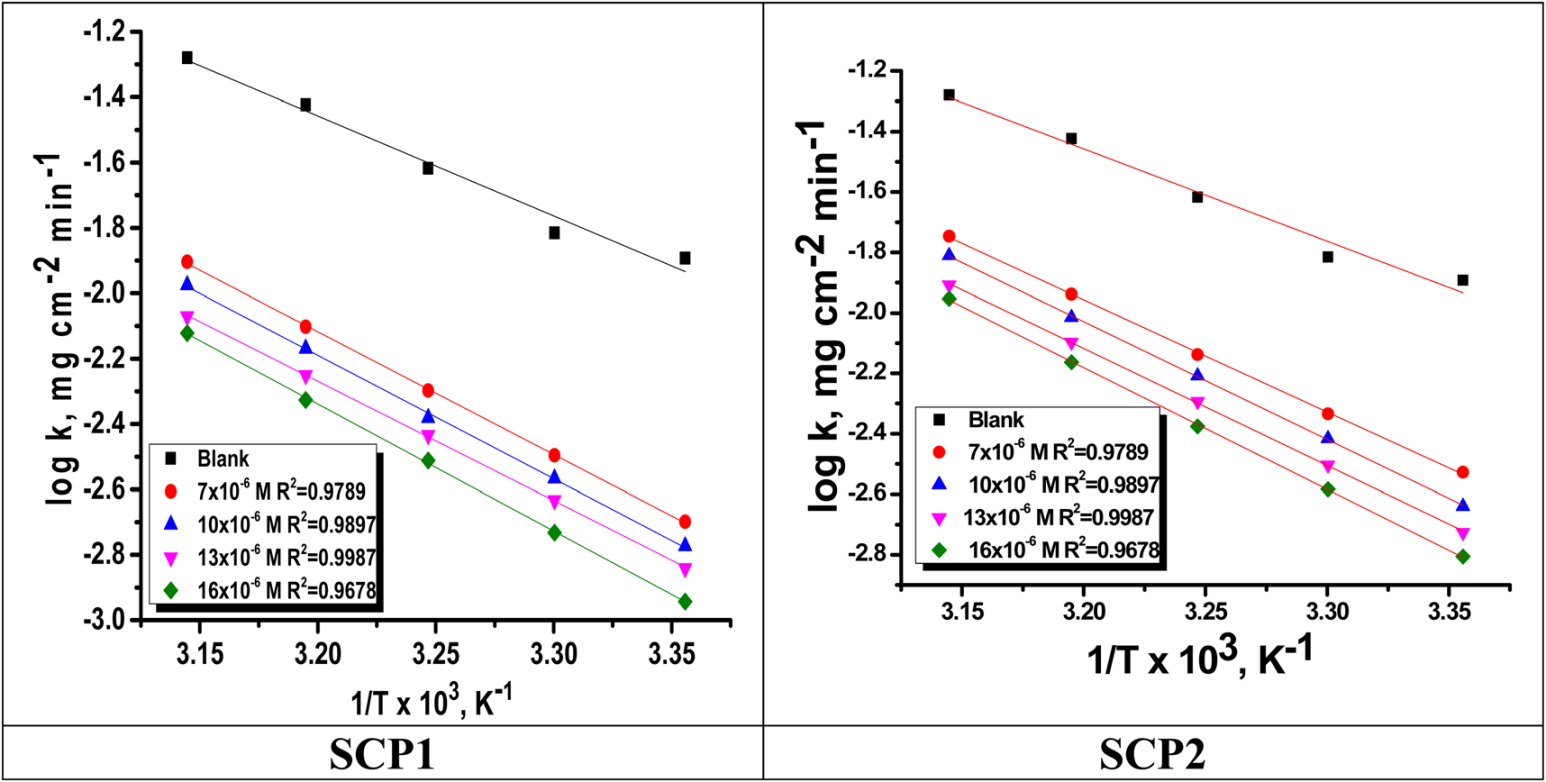
Arrhenius bends for SCPs inhibitors corrosion in 1.0 M HCl solution and with: SCP1 and, SCP2.

The relation between the *k* (corrosion rate) and temperature is expressed by the Arrhenius equation, eqn (7):^40^7
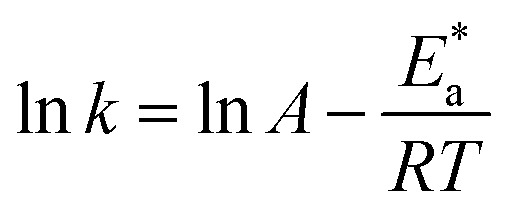
where 
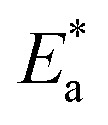
 is the activation energy. The plots of ln *k vs.* 1/*T* are illustrated in Fig. 4, from this plot, the values of 
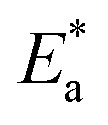
 were computed and are inserted in Table 5. The increased 
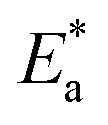
 values in the presence of SCP1 and SCP2 were greater than that obtained in the blank. These findings indicate the adsorption of SCP1 and SCP2 on the SS304 surface, constructing a barrier to separate such surface from the corrosive solution.^41^ By stopping the charge/mass transfer interaction on the surface, the adsorbed layer shields the SS304 from strong acid assault.^42^

**Table 5:** Activation parameters of the dissolution of SS304 in 1.0 M HCl with and without SCPs at 25–45 °C

Inhibitor	Conc., ×10^6^ M	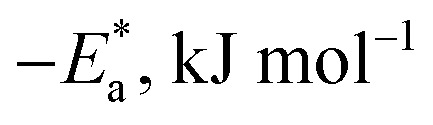	ΔH*, kJ mol^−1^	−ΔS*, J mol^−1^ K^−1^
Blank	0.0	66.4 ± 0.2078	63.9 ± 0.2071	78.2 ± 0.1341
SCP1	7	78.1 ± 0.1202	75.6 ± 0.0882	52.7 ± 0.1453
	10	79.8 ± 0.0882	77.3 ± 0.1528	53.8 ± 0.1202
	13	81.1 ± 0.2603	78.6 ± 0.2028	52.9 ± 0.2028
	16	82.9 ± 0.2887	80.4 ± 0.1202	59.1 ± 0.1528
SCP2	7	76.9 ± 0.2309	74.4 ± 0.1155	53.9 ± 0.2028
	10	78.2 ± 0.1528	75.7 ± 0.1764	54.6 ± 0.2082
	13	78.8 ± 0.1856	76.3 ± 0.2404	42.9 ± 0.1764
	16	81.1 ± 0.1732	78.6 ± 0.1732	48.6 ± 0.2404

The values of 
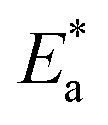
 were smaller than 80 kJ mol^−1^ that required for chemical adsorption, indicating that the kind of adsorption was physical.^43^ The dissolution of SS304 shows endothermic properties, as indicated by the positive ΔH* readings, suggesting that the presence of SCPs reduces the corrosion rate.

The outcome data (ΔH* and ΔS*) are evaluated *via*eqn (8):^44,45^8
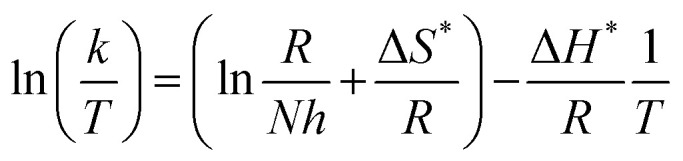


**Fig. 5:**
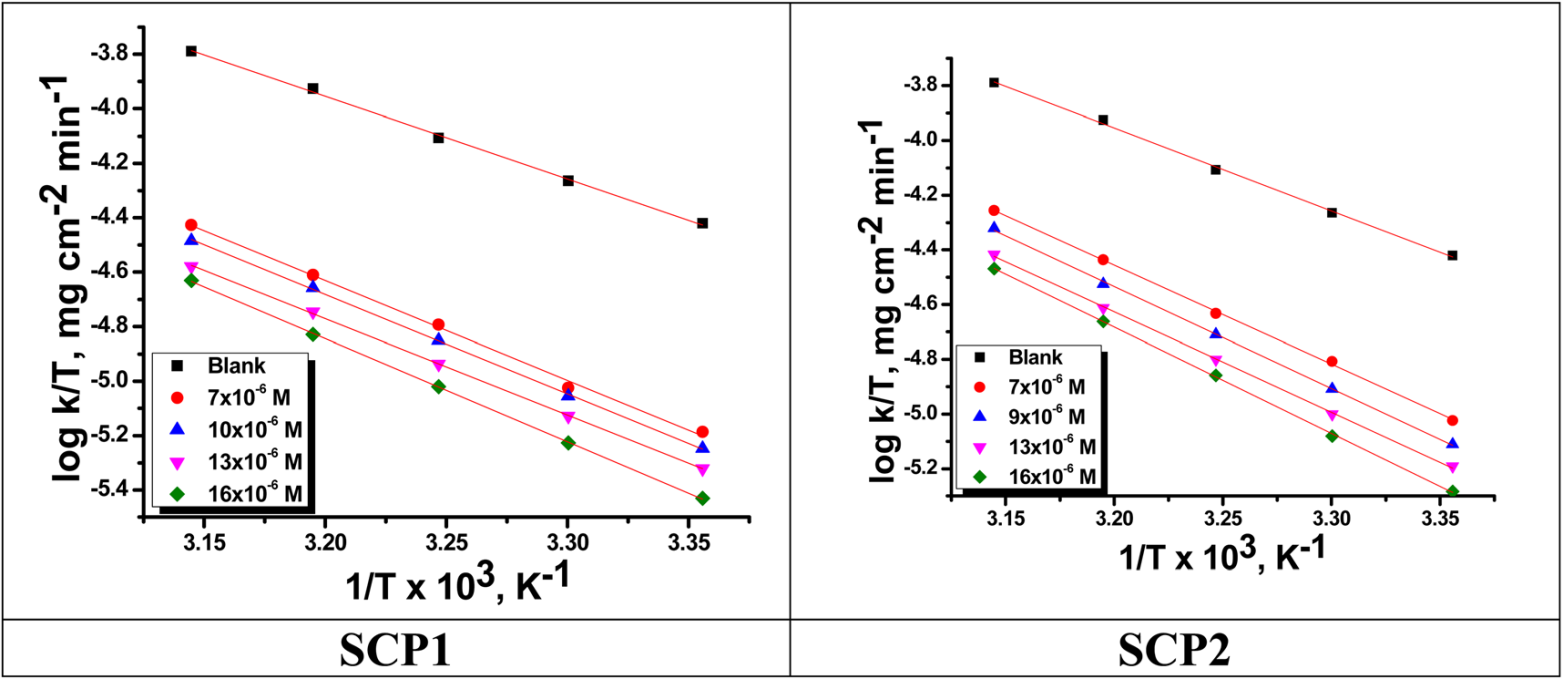
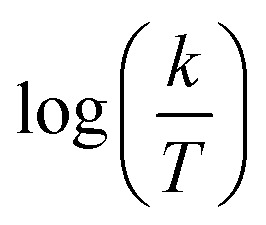
 *vs.* 
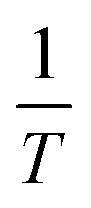
 plots for SCPs inhibitors corrosion in 1.0 M HCl solution and with: SCP1 and, SCP2.

The plots of 
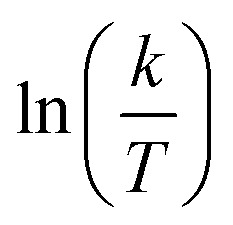
*vs.*
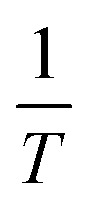
 were set to straight Fig. 5. The evaluated values of ΔH* and ΔS* are listed in Table 6. The positive ΔH* values obtained suggest that the dissolution process of SS304 is endothermic in nature and the ΔH* values increase with increasing SCPs concentration, suggesting that the dissolution reaction of SS304 to a higher concentration requires more energy. The presence of SCPs in the inhibited solution reduces the freedom of motion of inhibitor molecules, as shown by the negative activation entropy (ΔS*) signals. The activated complex also exhibits a association rather than dissociation in the rate-determine step, demonstrating the occurrence of a successive arrangement from the transition of the reactants to the activated complex.^46,47^ The results obtained allow us to verify the well-known thermodynamic relationship between 
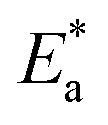
 and ΔH* (eqn (9)), which characterizes a monomolecular reaction:^48^9
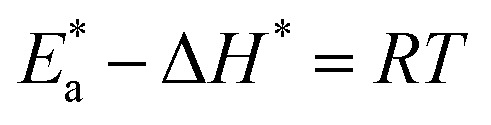


**Table 6:** PDP data for the dissolution of SS304 in 1.0 M HCl solution in the absence and presence of the SCPs inhibitors at 25 °C

Inh.	[Inh.] × 10^6^ M	−*E*_corr_, mV	*i*_corr_, μA cm^−2^	−*β*_c_, mV dec^−1^	*β*_a_, mV dec^−1^	*k*, mm	*θ*	*η* (%)
SCP1	0.0	138 ± 0.2431	795 ± 0.1732	133	63	415	—	—
	7	131 ± 0.2431	201 ± 0.2028	124	57	293	0.747	74.7
	10	126 ± 0.1742	158 ± 0.1155	143	51	231	0.801	80.1
	13	118 ± 0.2102	142 ± 0.2603	121	41	208	0.821	82.1
	16	128 ± 0.2010	79 ± 0.1764	127	45	116	0.901	90.1
SCP2	7	132 ± 0.1753	235 ± 0.2028	101	60	342	0.704	70.4
	10	122 ± 0.1208	163 ± 0.1732	126	52	237	0.795	79.5
	13	117 ± 0.2128	149 ± 0.1453	154	58	218	0.813	81.3
	16	130 ± 0.2127	141 ± 0.1732	116	50	205	0.823	82.3

The calculated value (2.50 at 298 K) is too close to the value estimated in Table 6. The inhibitor therefore acts on 
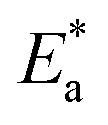
 and ΔH* in the same way.

### 3.4 Electrochemical analysis

#### 3.4.1 PDP studies

**Fig. 6:**
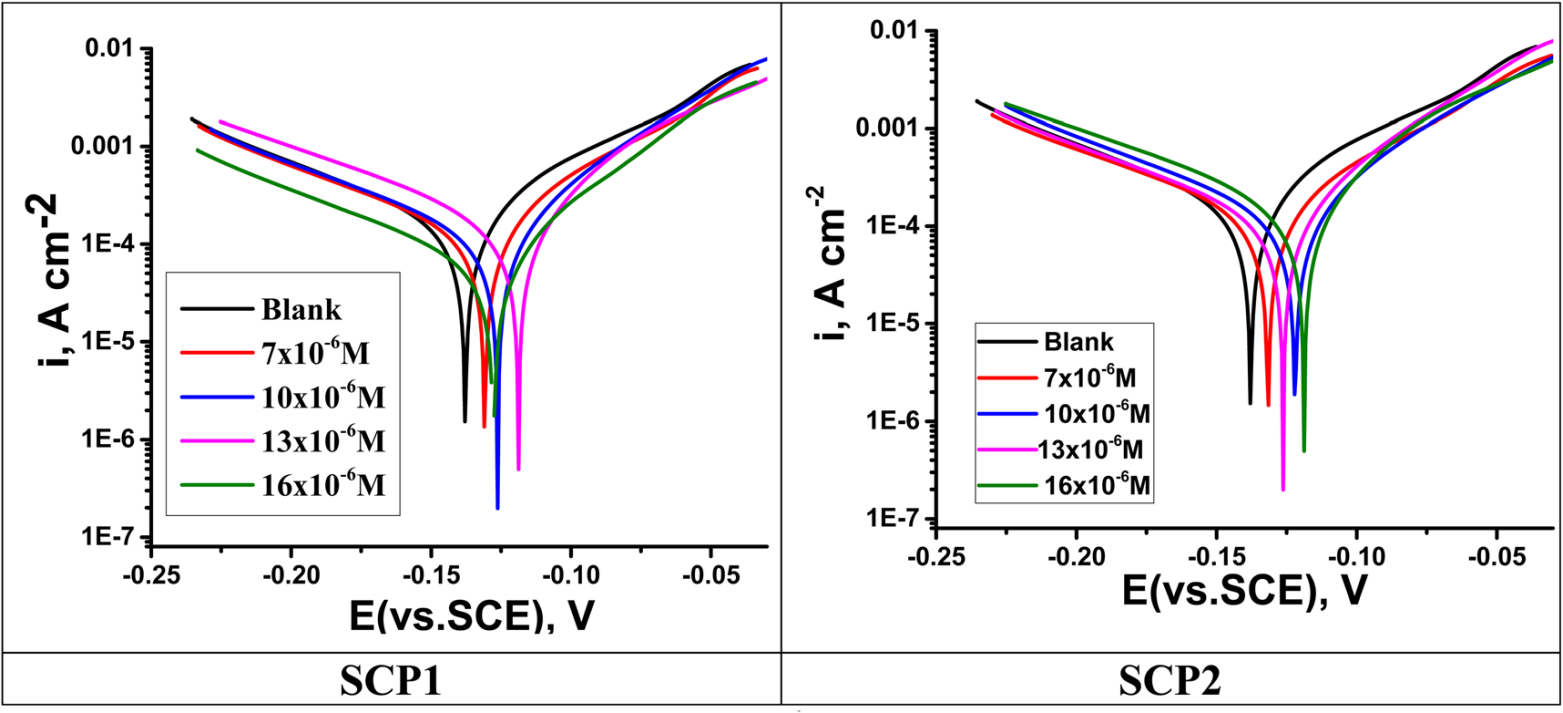
PDP curves for SS304 obtained at 25 °C in 1.0 M HCl solution containing altered concentrations of SCP1 and SCP2.

A plot of the PDP tests was used to determine the inhibitory mechanisms of SCPs compounds. SCP1 and SCP2 concentrations were determined using Tafel polarization curves, which are shown in Fig. 6. The deduced electrochemical parameters from Tafel plots such as corrosion potential (*E*_corr_), Tafel slopes (*β*_c_ and *β*_a_), corrosion current density (*i*_corr_), and the corresponding inhibition efficiency (*η*_Tafel_) are shown in Table 6. Polarization curve analysis shows that the introduction of SCPs inhibitors into the 1.0 M HCl solution resulted in lower anodic and cathodic current densities for both anodic and cathodic processes. Therefore, the dissolution reactions in the anodic branch are delayed, hydrogen gas accumulates when the cathodic branch is activated, and SS304 corrosion is inhibited. The cathode-panel lines in Fig. 6 are formed in parallel, indicating that the addition of SCPs has no effect on the hydrogen evolution mechanism, and the reduction of hydrogen ions on the surface of SS304 is mainly through the hydrogen transfer mechanism charge.^49^ According to this study, the absence of important fluctuations in *E*_corr_ values in the presence of SCPs compared to *E*_corr_ in the absence of SCP (10–17) mV indicates that SCPs are mixed inhibitors (anode–cathode).^50^ As a result, the cathodic evolution of hydrogen was not significantly affected by the adsorbed molecules. Based on these results, it appears that increasing inhibitor concentration decreases current density and thus, corrosion rate due to the adsorption of SCPs molecules on SS304 surface, consequently enhances the *η*%. These data suggest that an inhibitory effect exists.

#### 3.4.2 EIS studies

**Fig. 7:**
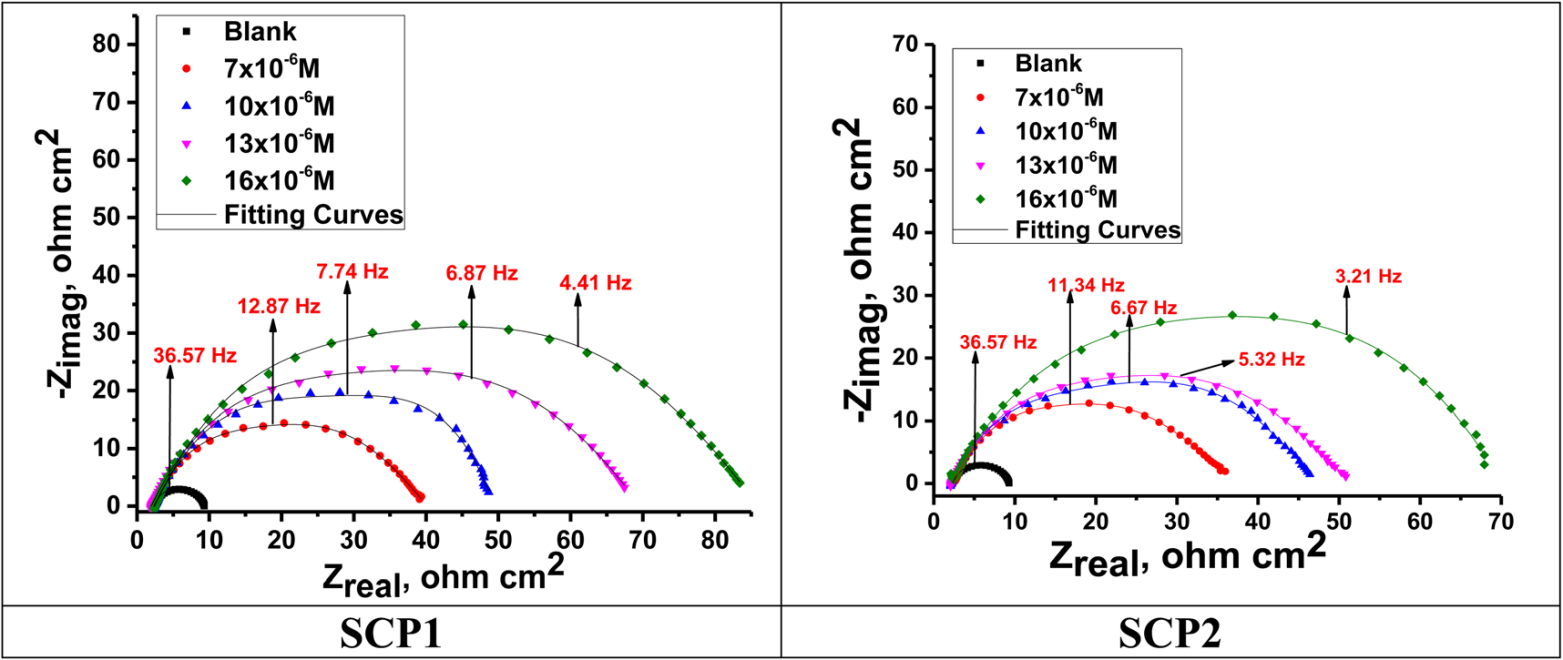
Nyquist bends for SS304 in 1.0 M HCl without and with altered concentrations of inhibitors SCP1 and SCP2 at 25 °C.

**Fig. 8:**
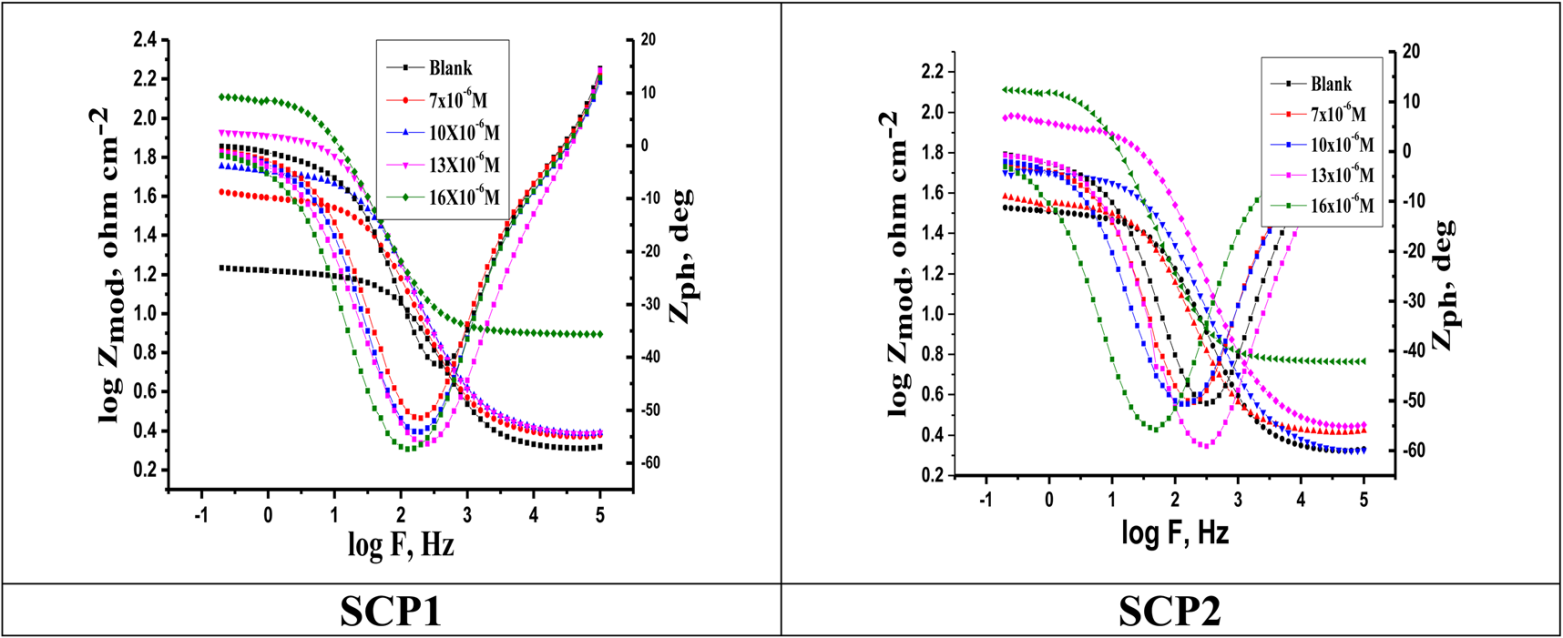
Bode plots for SS304 in 1.0 M HCl attendance and without altered concentrations of inhibitors SCP1 and SCP2 at 25 °C.

For the analysis of electrochemical systems, EIS stands out as one of the most useful techniques (EIS). The Nyquist and Bode plots shown in Fig. 7 and 8, respectively illustrate surface characteristics of SS304 samples and dynamics electrochemical processes in an uninhibited solution of 1.0 M HCl, and at various concentrations of SCP1 and SCP2. A charge transfer process is implied by the half-loop Nyquist curves of the studied system with one time constant. Blank loops have a smaller diameter than inhibitor loops, and loop forms are not altered as inhibitor concentration increases. According to these results, the corrosion process is not modified, and a covering film may form on the surface of the stainless steel due to adsorption of inhibitors.^51^ Thus, corrosion reactions are governed by the process of charge transfer between metals and solutions. The electrode surface may be protected from acidic solution by an adsorbed inhibitor film (SCP1 and SCP2), which slows contact with the acidic solution and inhibits SS304 dissolution. An evident time constant is observed in the Bode diagram Fig. 8. A frequency dispersion induced deviation has been avoided by replacing the ideal capacitance of double layer (*C*_dl_) with the CPE in the equivalent circuit”. This formula (eqn (10)) defines the CPE parameter as the flattened nature of Nyquist spectra allows us to have a more precise fit.^52^

**Fig. 9:**
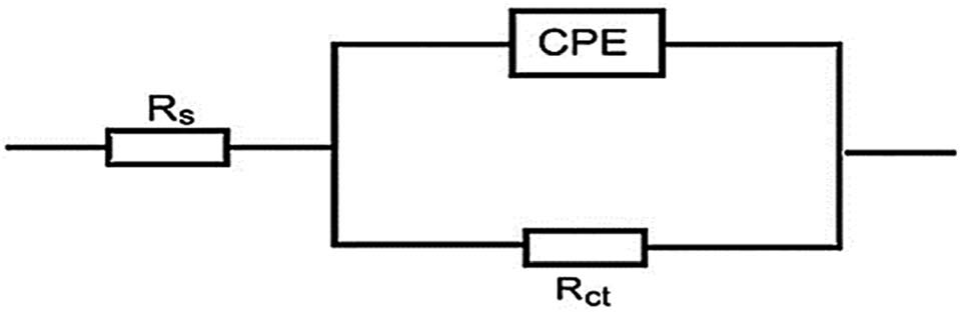
Electrical circuit for experimental data fitting of SCP1 and SCP2.

10 *Z*_CPE_ = *Q*^1−^ × (i × *ω*)^−1^

where the parameters (*Q*, i, *ω*, *n*) are respectively in this order, CPE constant, imaginary number with i^2^ = −1, the angular frequency with *ω* = 2 × π × *f* and CPE exponent lies within a range of −1 to 1. CPE in Fig. 9 explains the circuit components based on the value *n*. Thus, for exponent value *n* equal to 1, 0, 0.5, or 1, the following values are given: inductance, resistance, and Warburg impedance”. *n* is a measure that reveals a deviation from the ideal behaviour. The following criteria were used to determine which SCPs suited best: the tolerated errors of the elements in fitting mode (5%), as well as the chi-square error, were both small (*χ*^2^ < 10^−3^). Table 7 lists the SCPs parameters' numerical values. The *Y*_0_ estimate for the reference electrolyte is higher than that for the inhibited electrolyte. This suggests that SCPs molecules interact with the electrode surface, thereby limiting the destruction of exposed electrode sites. The next formula ( eqn (11)) was used to calculate the double layer capacitances, *C*_dl_, of a circuit containing a CPE.^53^

11 *C*_dl_ = (*Q* × *R*_p_^1−*n*^)^1/*n*^

where, *Q* is the magnitude of the CPE, i indicate the imaginary number of CPE, *ω* is the angular frequency (*ω*_max_ = 2π*f*_max_), *f*_max_ is the maximum frequency, and *n* is the empirical constant. In fact, the increase in *R*_p_ value and the concomitant decrease in *C*_dl_ with the increase in SCPs concentration suggest that the corrosive ions and water molecules coming from the surface of the substrate are replaced by inhibitory molecules, which increases the thickness by double electrical power layer and reduces the local dielectric constant and this is a sign that SCPs were acting at the SS/acid interface.^48^ However, the increase in *n* value after the addition of SCP in the 1 M HCl electrolyte is larger than that in the reference electrolyte, which can be interpreted as a certain reduction in surface heterogeneity.^54^ In uninhibited and inhibited solutions, the polarization resistance *R*_p_ can be used to calculate SCPs inhibition efficiency (eqn (12)).^55^12
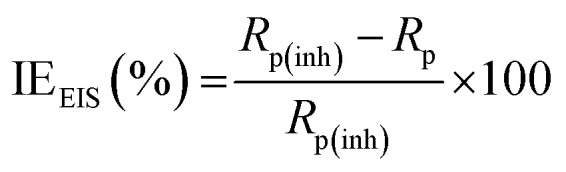


**Table 7:** EIS for SS304 after immersion in 1.0 M HCl in the presence of altered concentrations of SCP1 and SCP2

Comp.	Conc., ×10^6^ M	*R*_ct_, Ω cm^2^	*C*_dl_, ×10^4^, μF cm^−2^	*n*	*Y*_0_, (μΩ^−1^ s^*n*^ cm^−2^) × 10^−6^	*Θ*	*η* (%)	Goodness of fit *χ*^2^
Blank	0.0	6.7 ± 0.1456	147 ± 0.2775	0.915	164	—	—	8.55 × 10^−3^
SCP1	7	40.8 ± 0.2516	122 ± 0.2309	0.939	152	0.836	83.6	6.57 × 10^−3^
	10	48.8 ± 0.1527	104 ± 0.1201	0.948	145	0.863	86.3	6.54 × 10^−3^
	13	63.2 ± 0.2728	78 ± 0.2333	0.958	115	0.894	89.4	5.74 × 10^−3^
	16	86.9 ± 0.2333	67 ± 0.1855	0.959	105	0.923	92.3	4.89 × 10^−3^
SCP2	7	37.3 ± 0.1808	131 ± 0.2496	0.913	160	0.820	82.0	4.32 × 10^−3^
	10	43.1 ± 0.1201	118 ± 0.1732	0.914	151	0.845	84.5	5.77 × 10^−3^
	13	47.9 ± 0.2309	86 ± 0.1527	0.921	126	0.860	86.0	7.12 × 10^−3^
	16	64.7 ± 0.1763	71 ± 0.2185	0.936	113	0.896	89.6	6.66 × 10^−3^

Table 7 displays the values of the EIS's fitted parameters. SCP1 and SCP2 increase the *R*_ct_ much more than uninhibited solutions. Observations suggest that SCP1 and SCP2 molecules adhere to SS304 surfaces, forming a protective layer.^56^ The outcomes from Table 7 indicate a further increase in inhibition efficiency was observed for both SCP1 and SCP2, respectively, at 92.3% and 89.6%.

### 3.5 Theoretical analysis

#### 3.5.1 Quantum chemical parameters

**Fig. 10:**
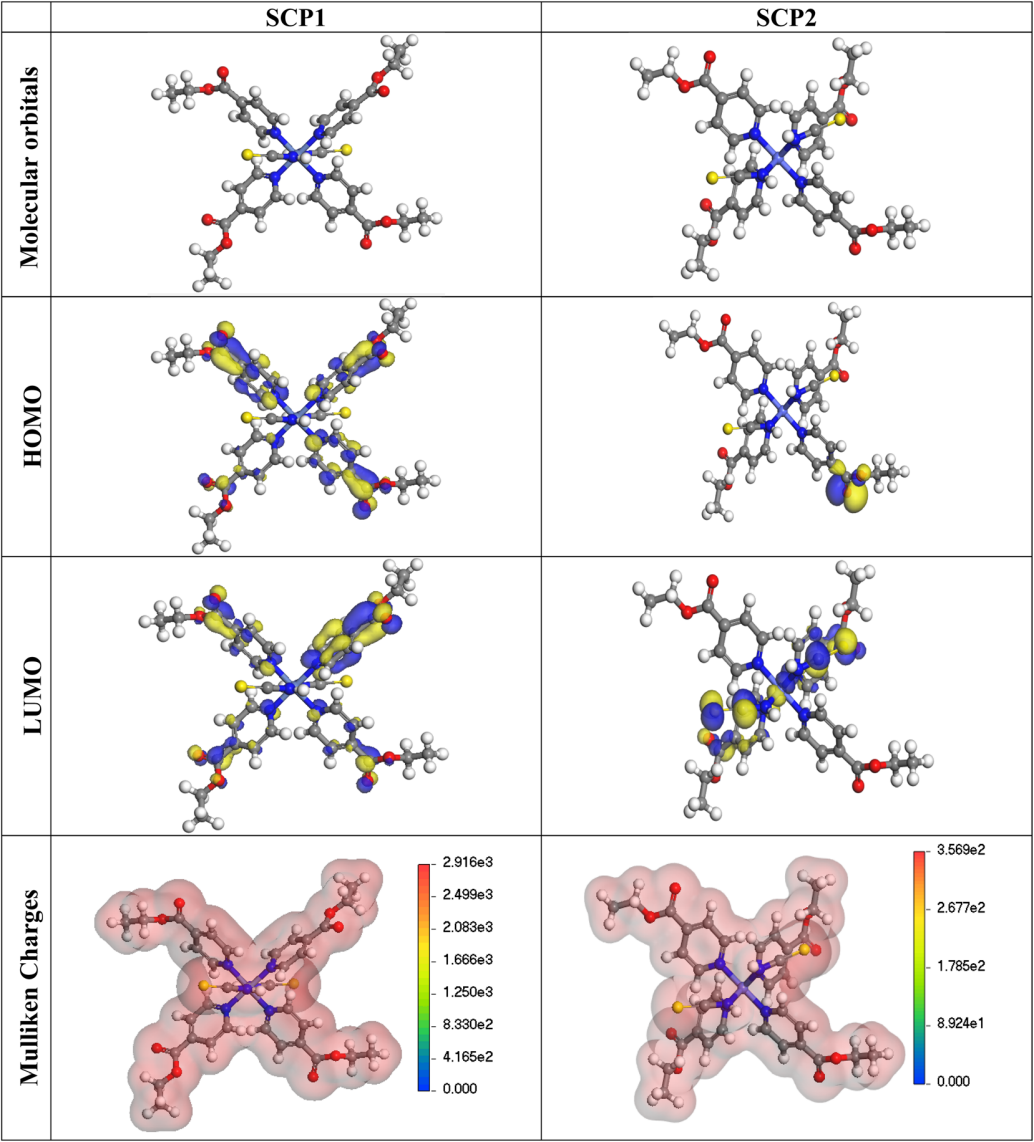
The optimized geometrical structure, (HOMO), and (LUMO) of the tested SCPs at DMol3.

The lower energy band gap value, which is represented in the energy band gap Δ*E*_g_ (Δ*E* = *E*_HOMO_ − *E*_LUMO_), indicates that organic molecules are highly reactive and exhibit excellent corrosion behaviour on the surface of SS304. An analysis of the impact of SCPs molecule's orientation on inhibition performance was conducted using density functional theory (DFT). As shown in Fig. 10, the optimized geometry, HOMO surface, and LUMO surface of studied inhibitors can be found. The parameters HOMO (*E*_H_), LUMO (*E*_L_), and dipole moment (*μ*) for MOFs gradients were directly obtained from DFT (Table 8). Eqn (13)–(18) were used to calculate the energy gap (Δ*E*), electronegativity (*χ*), global hardness (*η*), global softness (*σ*), the fraction of electron transfer (Δ*N*) and back-donation (Δ*E* back-donation)”, was calculated as Koopmans's theorem^57^ from the next balance:13
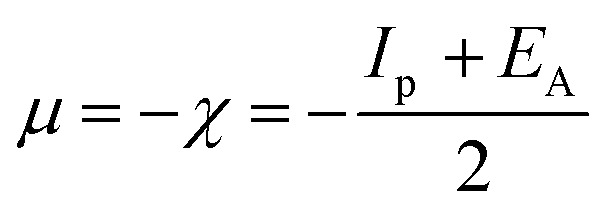
14
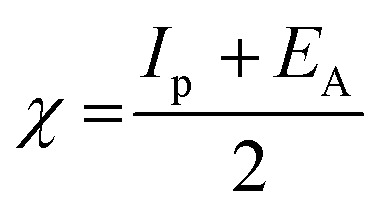
15
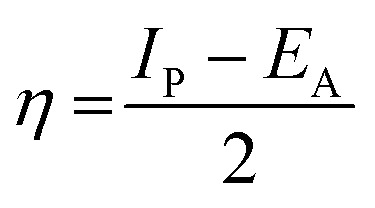
16
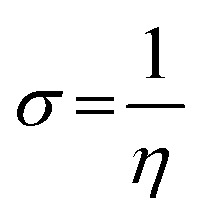
17
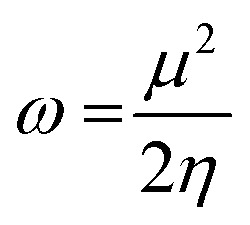
18
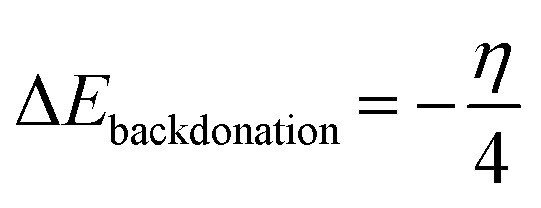


**Table 8:** Quantum chemical data for SCPs under study

Compound	SCP1	SCP2
−*E*_HOMO_, eV	4.999	6.027
−*E*_LUMO_, eV	3.968	4.183
Δ*E*, eV	1.031	1.844
*I*_P_, eV	4.999	6.027
*E*_A_, eV	3.968	4.183
*χ*, eV	4.4835	5.105
*η*, eV	0.5155	0.922
*σ*, eV	1.939864	1.084599
*ω*	19.49735	14.13288
Δ*N*	2.440834	1.027657
Δ*E*_back-donation_	−0.12888	−0.2305
Dipole moment (Debye)	12.87	18.53

Numerous articles^58,59^ have discussed how higher values of *E*_HOMO_ and lower values of *E*_LUMO_ determine the greater electron-donating and accepting abilities of an inhibitor. Inhibitors are more reactive when a lesser value of Δ*E* is present. In this instance, SCP1 Δ*E* value is lower while higher values for SCP2. In comparison to SCPs molecules, these values suggest that SCP1 molecule has a high degree of reactivity. Metals and inhibitors can be understood using the number/fraction of electron transfer (Δ*N*). If the Δ*N* value of an inhibitor is higher, it is found to have a stronger capability of donating electrons to metallic surfaces. Compared to SCPs molecules, SCP1 exhibits greater amounts of Δ*N* in the gaseous phase, indicating that SCP1 exhibits a stronger inhibitory effect.

#### 3.5.2 Monte Carlo (MC) simulation

**Fig. 11:**
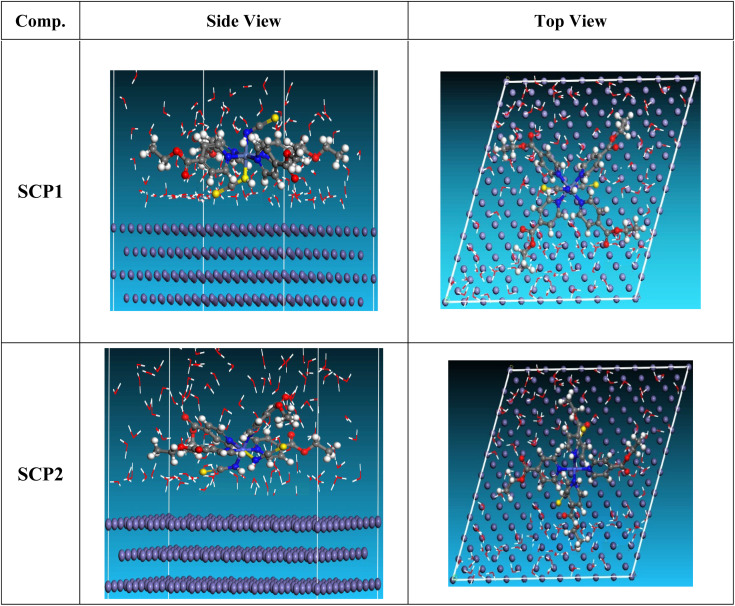
Adsorption shapes of the SCPs molecules on SS304 surface.

MC modeling is a good method for calculation the most stable adsorption conformations of a SCPs. Fig. 11 illustrates the simulation findings for the investigated SCP, which are described in Table 9. Fig. 11 depicts the adsorbed molecule's most favorable confirmation on the SS metal surface (111). Furthermore, the molecules stated are adsorbed on the metal surface from the motive, which is rich in inhibitory molecule electrons. The interactions between the occupied orbitals of the examined SCPs and the vacant orbitals of SS (111), which are reflected by energy adsorption values (*E*_ads_), of the rigid energy (*E*_rigid_), of the deformation energy (*E*_def_), and energy ratio values (d*E*_ads_/d*N*_i_) of the inhibitors, which is equivalent to the energy of substrate–adsorbate configurations where one of the adsorbate components has been removed are collected in Table 9. Adsorption energy values that are more negative indicate a highly stable and strong connection between adsorbed molecules and metal. When two materials are mixed during the adsorption process, an electron, ion, or molecule (adsorbent) is attached to the solid surface, adsorption energy is defined as declining energy.^60^ As shown in Table 9, the greater adsorption energy of SCP1 rather than SCP2 on the hardened Fe surface predicts heavy adsorption of SCP molecules, forming a stable adsorbed layer that protects the iron from decomposition. The tabulated adsorption energies are −3332.977 and −3178.027 kcal mole^−1^ for SCP1, SCP2 respectively. The outputs shows that the two inhibitors are efficient adsorptive inhibitors taking in respect that the better one is SCP1 which is attuned with the experimental results”. Based on theoretical modeling it's obvious that SCPs based proved to be powerful inhibitors for the SS304 which is confirmed by experimental and spectral investigation.

**Table 9:** MC parameters of adsorption of SCPs molecules on SS304 (111) surface

Structures	Adsorption energy	Rigid adsorption energy	Deformation energy	Compound d*E*_ad_/d*N*_i_	H_2_O d*E*_ad_/d*N*_i_
SS304 (111)/inhibitor SCP1/H_2_O	−3332.977	−3522.547	189.57	−265.58	−11.08
SS304 (111)/inhibitor SCP2/H_2_O	−3178.027	−3347.687	169.66	−223.79	−9.87

### 3.6 FT-IR spectroscopy

**Fig. 12:**
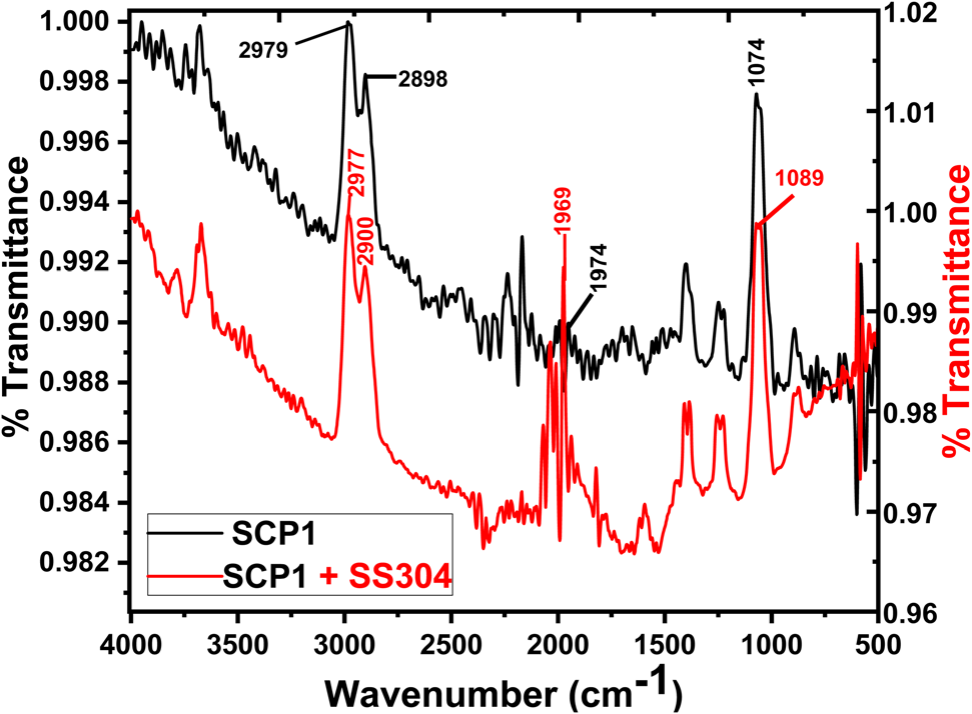
FTIR spectra of pure inhibitors and SS304 following a 24 hour exposure in an acidic environment with 16 × 10^−6^ M of SCP1 at 25 °C.

The inhibitors on the SS304 surface were detected by infrared spectroscopy in 1.0 M HCl in Fig. 12. On the surface of the SS304 specimen, the FTIR spectra of the pure inhibitors were compared to those of the adsorbed SCPs inhibitors. The inhibition spectrum and the adsorbed molecules of SCP1 on the surface of SS304 metal shows that certain peaks are moving or disappearing, while others become less prominent. This suggests the SCP1 compound is well absorbed by SS304 surfaces, which causes inhibition.^61^ At (2979, 2977) cm^−1^ are linked to the extension of chemical groups involving (–C–H) stretching group”. The spectral regions at (2898, 2900) cm^−1^ are linked to the extension of chemical groups involving (–CH_2_) aliphatic stretching group. Furthermore, the spectral feature at (1969, 1974) cm^−1^ is allocated to the stretching vibrations of specific (C=O) bonds. The observed peak at (1074, 1089) cm^−1^ indicates the presence of specific chemical functionalities, possibly related to (–C–N, –C–O) groups.

### 3.7 Characterization of the surface of SS304 (AFM analysis)

**Fig. 13:**
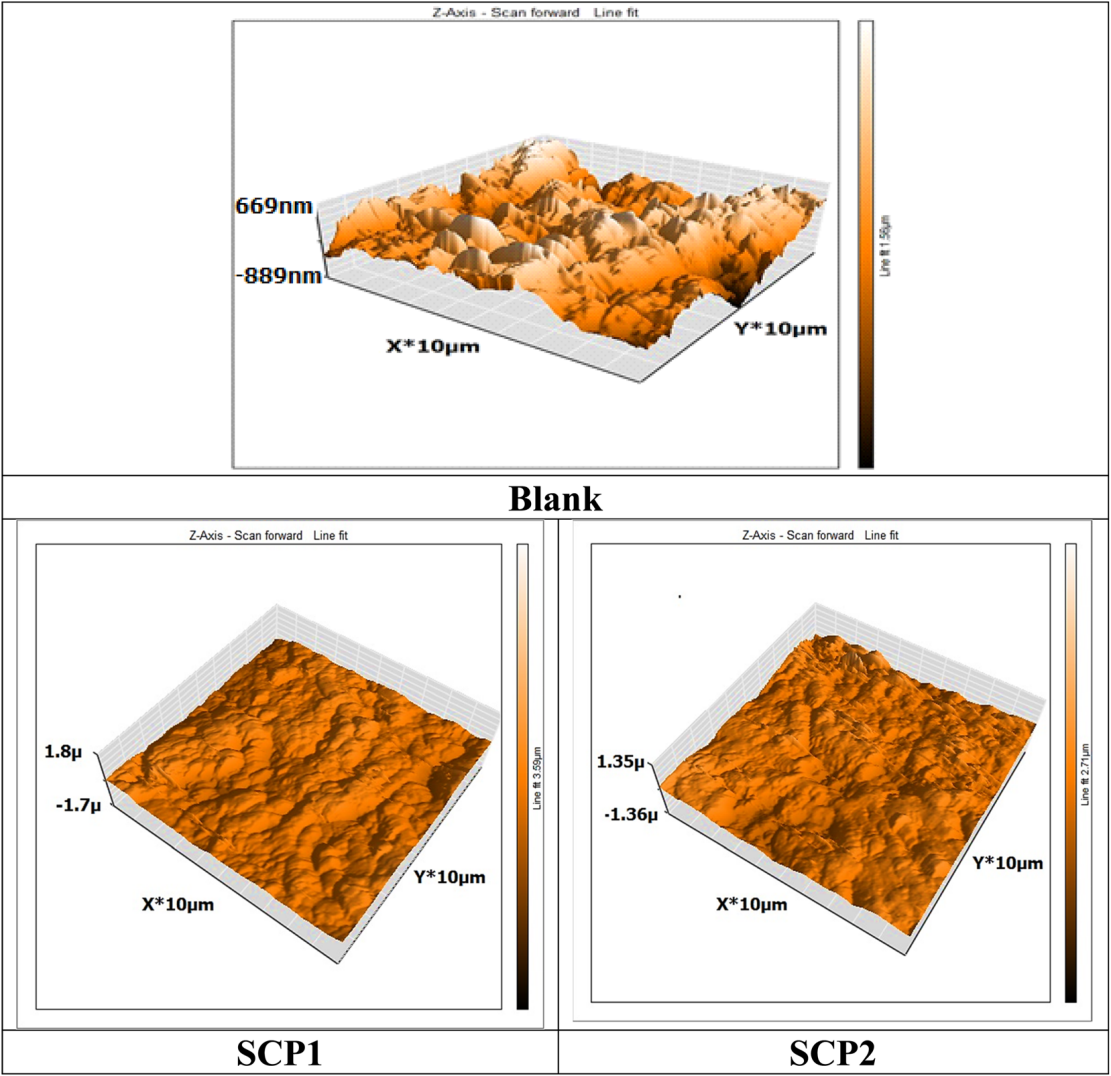
3D AFM morphology of SS304 samples surface without and with SCPs compounds.

An AFM analysis was conducted on the SS304 surface to check the existence of an inhibitor film. AFM images and force curves are shown in Fig. 13 after 24 hours of exposure in 1.0 M HCl solution presence and absence SCPs corrosion inhibitors. The mean roughness value of SS304 surface that was exposed to a 1.0 M HCl solution but was not treated with the inhibitor was substantially greater at 300 nm. The acid's corrosive effects over the course of 24 hour rust test period left the SS304 surface with a porous structure and deep fractures, which led to this heightened roughness. However, when the tested inhibitors are applied at the optimum concentration (16 × 10^−6^ M), the average roughness for SCP1 & SCP2 is reduced to 89 & 103 nm, respectively. The test inhibitors effectively maintain hardness of SS304, as seen by the drop in roughness value.^62^

### 3.8 Mechanism of adsorption and inhibition

**Fig. 14:**
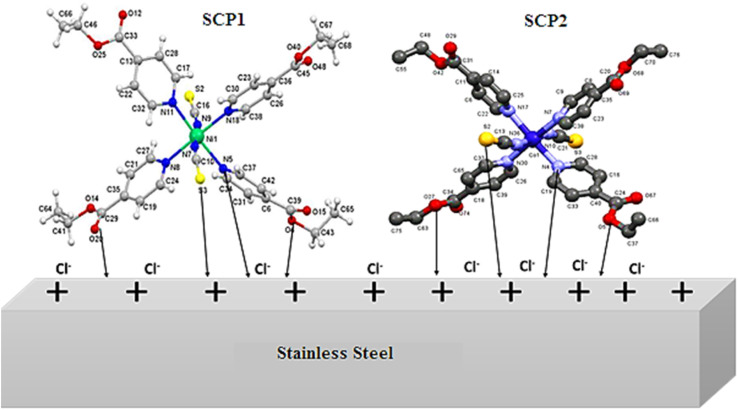
Mechanism of the adsorption of SCPs on SS304 surface.

The inhibition efficacy of SCPs compounds on SS304 in 1.0 M HCl can be understood well-known on the molecular size and interface modes of SCPs molecules on SS304 surface Fig. 14. The adsorption mechanism of the SCPs adsorbed on SS304 is generally due to chemical and physical adsorption. The reactive sites present in the molecular structure of the SCPs are responsible for the corrosion inhibition process that depends on the nature and the loading of the metal. The experimental and theoretical methods used in this work justified that the tested SCPs compounds are excellent corrosion inhibitors due to their ability to interact with the atoms of the SS304 surface. The presence of π-electrons and heteroatoms such as oxygen, aromatic unsaturated, and active functional groups promotes the corrosion inhibition of SS304.^63^ The interaction of the electron-rich aromatic ring with the unpaired electrons of the metal and the inhibitory actions of the SCPs molecules can be explained by the presence of electron-rich oxygen atoms.^64^ It is likely that various interactions can occur, namely: coordination of a metal atom with an unshared electron pair of the inhibitor molecules, sharing of π-electrons of the SCPs in the coordination process, and electrostatic attraction and/or interactions between the negatively charged metal surface and the positively charged SCPs molecules. The protonated form of the adsorbent molecules competes with the aqueous H^+^ ion. Nevertheless, upon the release of the H_2_ gas, the inhibitors return to their neutral form. The transfer of the unshared electron pairs is reproduced in the unoccupied d-orbital of the metal (retro-donation).^65^ However, this electron transfer generates the accumulation of an additional negative charge on the metal surface. Moreover, this will result in a fresh transfer (back-donation) to the inhibitor molecules' anti-bonding molecular orbitals.^66^

## 4. Conclusion

The main conclusions drawn from all these studies of SCPs compounds are: the *η*% of SCPs compounds improves with the rise in inhibitor concentrations whereas it lowered with the increase of temperature. The *η*% of all composites in altered methods followed the order: SCP1 > SCP2. The adsorption of all the composites on SS304 surface from the acidic solutions conform the Langmuir's adsorption isotherm. Polarization curves indicated that the SCPs act as mixed type inhibitor. SCPs increases *R*_ct_ values and decreases both *C*_dl_ and *i*_corr_ values in 1.0 M HCl solution. FTIR and AFM examination for SS304 surface revealed the attendance of a protective film, which protect SS304 *versus* the destructive media. The experimental finding agrees well with the theoretical calculations.

## Conflicts of interest

There are no conflicts to declare.

## Supplementary Material
